# Spectral Super-Resolution Technology Based on Fabry–Perot Interferometer for Temporally and Spatially Modulated Fourier Transform Imaging Spectrometer

**DOI:** 10.3390/s25041201

**Published:** 2025-02-16

**Authors:** Yu Zhang, Qunbo Lv, Jianwei Wang, Yinhui Tang, Jia Si, Xinwen Chen, Yangyang Liu

**Affiliations:** 1 Aerospace Information Research Institute, Chinese Academy of Sciences, No. 9 Dengzhuang South Road, Haidian District, Beijing 100094, China; zhangyu20e@mails.ucas.ac.cn (Y.Z.); lvqunbo@aoe.ac.cn (Q.L.); wjw@aoe.ac.cn (J.W.); tangyinhui18@mails.ucas.ac.cn (Y.T.); sijia@aircas.ac.cn (J.S.); chenxw@aircas.ac.cn (X.C.); 2 School of Optoelectronics, University of Chinese Academy of Sciences, No.19(A) Yuquan Road, Shijingshan District, Beijing 100049, China; 3 Department of Key Laboratory of Computational Optical Imagine Technology, Chinese Academy of Sciences, No. 9 Dengzhuang South Road, Haidian District, Beijing 100094, China

**Keywords:** fourier transform imaging spectroscopy, Fabry-Pérot interferometer, spectral super-resolution

## Abstract

A new spectral super-resolution technique was proposed by combining the Fabry–Perot interferometer (FPI) with Temporally and Spatially Modulated Fourier Transform Imaging Spectrometer (TSMFTIS). This study uses the multi-beam interference of the FPI to modulate the target spectrum periodically, and it acquires the modulated interferogram through TSMFTIS. The combined interference of the two techniques overcomes the limitations of the maximum optical path difference (OPD) on spectral resolution. FPI is used to encode high-frequency interference information into low-frequency interference information, proposing an inversion algorithm to recover high-frequency information, studying the impact of FPI optical defects on the system, and proposing targeted improvement algorithms. The simulation results indicate that this method can achieve multi-component joint interference imaging, improving spectral resolution by twofold. This technology offers advantages such as high throughput, stability, simple and compact structure, straightforward principles, high robustness, and low cost. It provides new insights into TSMFTIS spectral super-resolution research.

## 1. Introduction

Fourier transform imaging spectroscopy (FTIS) is based on the principle of two-beam interference, where the target spectrum undergoes optical Fourier transformation to obtain its interferogram [1]. Spectral information is obtained by performing inverse calculations on the interferogram. With the rapid development of spectroscopy, there is an increasing demand for the higher spectral resolution of interferometric spectrometers in various fields, such as atmospheric environment monitoring, water quality monitoring, and mineral exploration [2,3,4,5,6,7].

The spectral resolution of FTIS depends on the maximum optical path difference (OPD), while the step size of the OPD scan determines the spectral range. FTIS can be categorized based on the modulation method into Temporally Modulated FTIS (TMFTIS), Spatially Modulated FTIS (SMFTIS), and Temporally and Spatially Modulated FTIS (TSMFTIS) [1]. TMFTIS, due to the presence of moving mechanical components and its sensitivity to the tilt and lateral displacement of the moving mirror, find it challenging to achieve the large-stroke reciprocating motion of the moving mirror, thus having limited spectral resolution [8,9]. SMFTIS has a low light throughput due to the presence of a slit, and its optical path range is limited by the size of the image plane [1,10,11]. To improve light throughput, TSMFTIS was proposed based on the structure of SMFTIS, which features advantages such as high throughput, stable performance, and compact structure [11,12]. TSMFTIS is widely used in airborne or spaceborne remote sensing applications [13,14,15,16,17].

TSMFTIS generates interference fringes on the image plane through lateral shearing interferometry (LSI). Scanning the entire field of view along the direction of the vertical interference fringes allows for the interferogram to be obtained. Therefore, similar to SMFTIS, the range of TSMFTIS is limited by its image plane size [10,13,14]. When the spectral range is determined, TSMFTIS cannot directly increase lateral shear to extend the OPD. In recent years, research has improved the spectral resolution of static spectrometers; however, these systems do not capture spatial geometric information [18,19,20,21]. Currently, there is still a lack of relevant research to improve the spectral resolution of TSMFTIS.

The Fabry-Pérot interferometer (FPI) is based on the multi-beam interference principle and is characterized by its compact size, high transmittance, strong spectral splitting capability, and excellent structural stability. It is suitable for lightweight and high-precision hyperspectral imaging applications [22,23,24,25,26]. In addition to serving as the main spectral splitting components of the imaging spectrometer, some previous studies have integrated FPI into other imaging spectrometers to enhance their spectral resolution, including TMFTIR [27,28,29,30,31,32,33]. The principle is to use a tunable spaced FPI to generate extremely sharp comb-like transmission spectra, with each transmission peak finely sampled at different positions within the spectral imaging instrument’s spectral sampling interval. In theory, it is possible to achieve high-magnification spectral super-resolution. Theoretically, achieving high-magnification spectral super-resolution is possible. However, this requires a tunable FPI with a very high tuning precision. A tunable FPI has moving parts, which can reduce system stability and make it difficult to achieve a large aperture. This conflicts with the high stability and high light throughput of TSMFTIS. Most importantly, during the push-broom process of TSMFTIS, the FPI cannot be repeatedly tuned and sampled. Most importantly, during the push-broom process of TSMFTIS, where sampling is done pixel by pixel, the FPI cannot be tuned multiple times. Therefore, this method cannot be used to enhance the spectral resolution of TSMFTIS.

To enhance the spectral resolution of FTIS, we have, for the first time, proposed a multi-component joint interference spectral super-resolution technique based on fixed-interval FPI. This paper proposes a first-time TSMFTIS spectral super-resolution technique based on fixed-interval FPI, which we call Static Multi-Component Joint Interference Hyperspectral Imaging (SMJI-HI). It combines the multi-beam interference of FPI with the lateral double-beam interference of TSMFTIS. The role of FPI is to perform spectral mixing modulation on the target spectrum through multi-beam interference, while the role of TSMFTIS is to capture the modulated spectral information. Due to the modulation effect of the FPI, high-frequency interference information beyond the maximum OPD is encoded into low-frequency interference information and can be retrieved through algorithms. This process can be equivalently seen as extending the maximum OPD of TSMFTIS. In our previous research, we demonstrated the feasibility of achieving spectral super-resolution with TMFTIS [34]. In this paper, we focus on the joint interference of TSMFTIS and FPI, providing a detailed analysis of their joint interference principles, modulation principles, and the resulting interferograms, and we further demonstrate the feasibility of spectral super-resolution. In our previous research, we proposed the basic model for the inversion algorithm. Building on this foundation, this paper establishes an optimization model to reduce the ill-posedness of the algorithm and enhance the system’s robustness. The potential issues of OPD offset and frequency shift component broadening caused by optical defects of the fixed-interval FPI were analyzed. The inversion algorithm was further improved, along with improving algorithms for the real-time phase calibration and broadening compensation, enhancing the system’s robustness. This technology offers high stability, a simple and compact structure, low requirements for fixed-interval FPI specifications, low spectral recovery complexity, and high robustness. Compared to traditional TSMFTIS, the FPI does not require multiple tunings, and spectral super-resolution can be achieved with a single data acquisition. It is well-suited for TSMFTIS in terms of principles and data acquisition methods. Using a fixed-interval FPI makes it easier to achieve a large aperture and low mechanical tolerance, ensuring the light throughput and mechanical stability of TSMFTIS. Both FPI and TSMFTIS are based on interference principles and are well-matched in terms of field of view, spatial resolution, optical path difference, and optical structure.

## 2. Spectral Super-Resolution Principle Analysis of SMJI-HI Based on FPI

### 2.1. Principle Analysis of FPI

**Figure 1:**
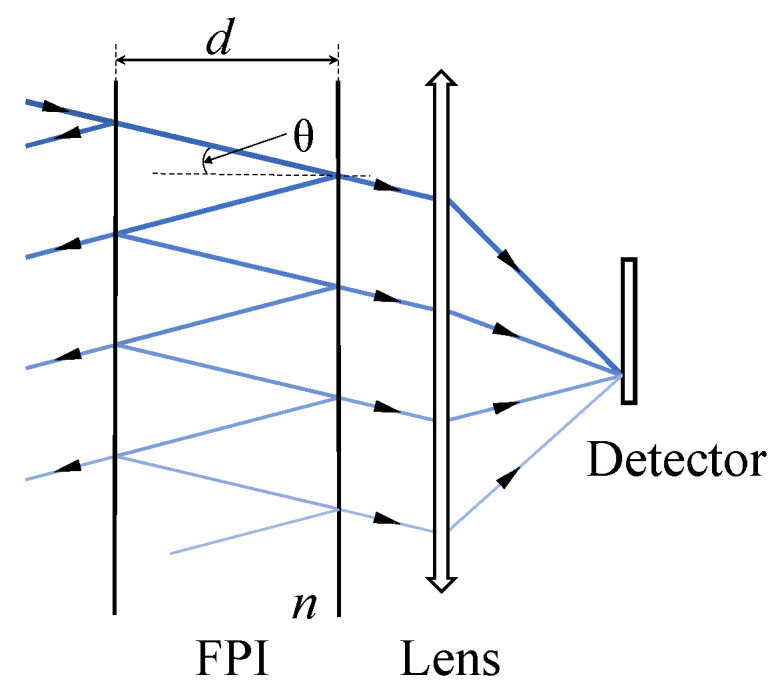
FPI principle schematic diagram.

FPI comprises two semi-reflective mirrors, with an interval of *d* and a medium refractive index of *n*. Its schematic diagram is shown in Figure 1. A beam of parallel light with wave number ν is incident on the FPI with an angle θ. After multiple reflections and transmissions, the outgoing light angle remains unchanged. The OPD between adjacent outgoing light beams is $\Delta_{FPI}=2nd\cos\theta$, and the phase difference is $\delta_{FPI}=4\pi \nu nd\cos\theta$.

The imaging system converges the FPI outgoing light beam on the image plane, resulting in multi-beam interference. The effect of interference can be regarded as producing a transmission spectrum, which is periodic in the wave number dimension. The transmission spectrum $T_{FPI}$ distribution is as follows:

(1) $$T_{FPI}\left(\nu \right)=\frac{{\left(1-R\right)}^{2}}{1+R^{2}-2R\cos\left(2\pi \nu \Delta_{FPI}\right)},$$

where *R* is the reflectivity of the FPI reflective surface. $T_{FPI}\left(\nu \right)$ is a periodic function of ν and can be expressed as a Fourier series, as follows [35]:

(2) $$T_{FPI}\left(\nu \right)=\sum_{m=0}^{\infty }a_{m}\cos\left(m\cdot 2\pi \nu \Delta_{FPI}\right).$$

Therefore, $T_{FPI}\left(\nu \right)$ comprises the fundamental wave with a periodic frequency of 2ndcosθ and its higher harmonics. When m=0, $a_{0}=\left(1-R\right)/\left(1+R\right)$; when m>0, $a_{m}=2R^{m}\left(1-R\right)/\left(1+R\right)$. Therefore, $a_{m}$ depends only on *R* [35].

### 2.2. Principle Analysis of SMJI

**Figure 2:**
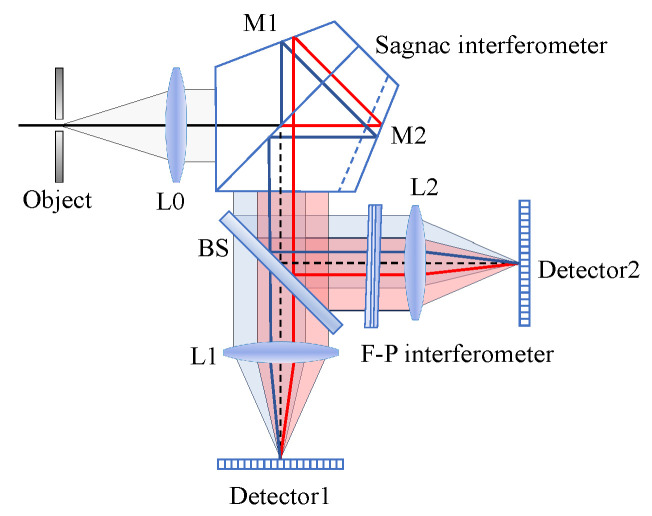
Schematic diagram of system structure.

The schematic diagram of the SMJI-HI system based on FPI and TSMFTIS is shown in Figure 2. First, the object beam passes through the collimation system L0 and enters the LSI, and the outgoing beam is divided into two paths by the beam splitter. The LSI takes the Sagnac interferometer as an example. One beam passes through the imaging system L1 and converges on Detector1; the other beam passes through the FPI and then passes through the imaging system L2 and converges on Detector2. The imaging system and detector are the same in the two optical paths, and the only difference is whether there is FPI. On Detector1, the interferogram $I_{1}$ from the Sagnac interferometer can be obtained; on Detector2, the interferogram $I_{2}$ resulting from the joint interference of the Sagnac interferometer and the FPI can be obtained. The maximum OPD of the Sagnac interferometer determines the maximum OPD of these two interferograms. Subsequently, based on the designed algorithm, a new interferogram with double the OPD is calculated by combining the two interferograms, thereby achieving spectral super-resolution.

#### 2.2.1. Optical Path Without FPI

**Figure 3:**
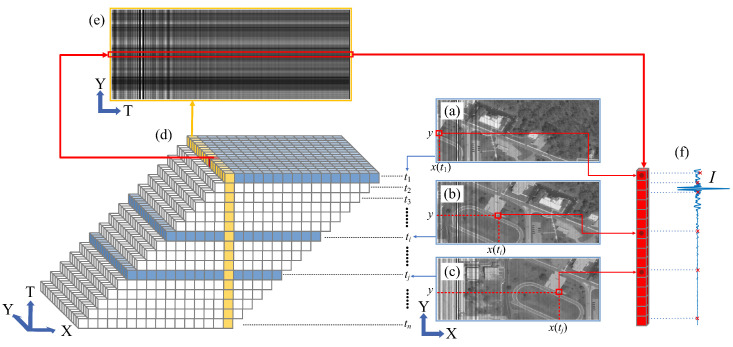
Schematic diagram of teh TSMFTIS interferogram scanning method: (**a**–**c**) interference images captured by TSMFTIS at times $t_{1}$, $t_{i}$, and $t_{j}$, where the position of a specific target point in the image is $\left(x\left(t_{1}\right),y\right)$, $\left(x\left(t_{i}\right),y\right)$, and $\left(x\left(t_{j}\right),y\right)$, respectively; (**d**) pushing the scan along the X direction, interference data are assembled based on the actual spatial positions and time sequence; (**e**) interference patterns formed by the Y dimension of space and the T dimension of time; (**f**) The interferogram of a specific target point formed by assembling data in time sequence.

The LSI generates two beams with a certain optical path difference. After passing through L1, they are imaged on Detector1, and double-beam interference occurs. The interferogram of Detector1 is the same as that of the traditional TSMFTIS, as follows:

(3) $$I_{1}\left(x,y,\nu ,\Delta \right)=R_{sag}T_{sag}B_{x,y}\left(\nu \right)\left[1+\cos\left(2\pi \nu \Delta \right)\right],$$

where $R_{sag}$ and $T_{sag}$ are the reflectivity and transmittance of the beam splitter of the Sagnac interferometer respectively, Δ=Dx/f is the OPD produced by the Sagnac interferometer, *D* is the transverse shear amount, $B_{x,y}\left(\nu \right)$ is the corresponding object point spectrum at the detector (x,y) position, and *f* is the focal length of L1 and L2. Equation (3) describes the interference images collected by the Sagnac interferometer at a certain moment, corresponding to Figure 3a–c. Therefore, each column of pixels along the X direction on the image surface has a fixed and known OPD, forming longitudinal interference fringes, as shown in Figure 3a–c. The target scene is completely scanned across the image plane along the interference direction X by push scanning. Data are collected every time the scene moves one pixel. The interference signals at all pixels in the X direction for the same target point are obtained over the times $t_{1}$∼$t_{n}$. All the collected interference images are then combined according to the target’s real-world spatial coordinates and time, as shown in Figure 3d. By extracting all the interference data along the T direction for the target point located at (X, Y), these data form the interferogram for that target point, as shown in Figure 3f. Through continuous scanning, the interferograms for all objects in the scene can be obtained. This is the working principle of TSMFTIS.

The interferogram of the target is obtained after push scanning. Based on Equation (3), after ignoring the coefficient and removing the DC component, the expression of the monochromatic light interferogram is as follows:

(4) $$I_{1}\left(\nu ,\Delta \right)=B\left(\nu \right)\cos\left(2\pi \nu \Delta \right).$$

The interferogram of polychromatic light is as follows:

(5) $$I_{1}\left(\Delta \right)=\int B\left(\nu \right)\cos\left(2\pi \nu \Delta \right)d\nu =\mathrm{FT}\left[B\left(\nu \right)\right],$$

where FT denotes the Fourier Transform. In traditional TSMFTIS, the spectrum can be directly obtained by the inverse Fourier transform (IFT) of $I_{1}\left(\Delta \right)$. However, the OPD is limited, and the maximum OPD depends on the amount of lateral shear and the size of the image plane, which is considered fixed and known in this study. Assuming the maximum OPD is *L*, the high-frequency interference information of $I_{1}\left(\Delta \right)$ outside *L* will be lost, and the spectral resolution of TSMFTIS is limited.

#### 2.2.2. Optical Path with FPI

**Figure 4:**
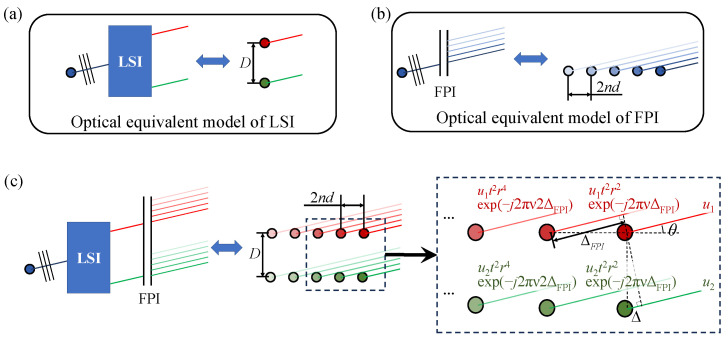
(**a**) Optical equivalent model of LSI; (**b**) Optical equivalent model of FPI; (**c**) optical equivalent model of SMJI between the LSI and FPI.

Due to the addition of FPI, another path must consider the lateral shear double-beam interference and multi-beam interference joint interference. As shown in Figure 4, the principle of the LSI can be equivalent to shearing the incident parallel light into two components. The lateral shearing amount is *D*. The working principle of FPI can be equivalent to generating countless components longitudinally from the incident parallel light. The energy decreases step by step, and the longitudinal interval is $\Delta_{FPI}=2nd$. Therefore, the joint interference of the two can be regarded as shearing into two components in the horizontal direction and forming countless components in the vertical direction.

Assume that a parallel beam of wave number ν is incident and its complex amplitude is *u*. After passing through the transverse shearing interferometer, ignoring the coefficient, the complex amplitudes of the two outgoing parallel light beams are $u_{1}=u$ and $u_{2}=u\exp\left(-j2\pi \nu \Delta \right)$, where *j* is an imaginary unit. The complex amplitudes of the beams after passing through the FPI are shown in Figure 4c, where *r* and *t* are the reflection and transmission coefficients of the FPI, respectively. The outgoing beams of the LSI and FPI are parallel beams with the same angle. All the outgoing beams are converged and interfered with at the same time, and the total complex amplitude is

(6) $$U=ut^{2}\left[1+\exp\left(-j2\pi \nu \Delta \right)\right]\sum_{i=0}^{\infty }{\left[r^{2}\exp\left(-j2\pi \nu \Delta_{FPI}\right)\right]}^{i}.$$

The interference light intensity for a single wavelength is as follows:

(7) $$I_{2}=UU^{*}={\left|u\right|}^{2}{\left|[1+\exp(-j2\pi \nu \Delta )]\right|}^{2}\left|\sum_{i=0}^{\infty }t^{2}{\left[r^{2}\exp\left(-j2\pi \nu \Delta_{FPI}\right)\right]}^{i}\right|.$$

Therefore, the monochromatic light interference intensity can be written as the product of the three terms in Equation (7). The first term is the incident light intensity $B\left(\nu \right)={\left|u\right|}^{2}$. The second term is the interference transmission of the Sagnac interferometer, where the forward coefficient is neglected, resulting in $\left[1+\cos\left(2\pi \nu \Delta \right)\right]\propto {\left|1+\exp(-j2\pi \nu \Delta )\right|}^{2}$. The third term is the FPI transmission, which is the same as in Equation (2), $T_{FPI}\left(\nu \right)={\left|\sum t^{2}{\left[r^{2}\exp\left(-j2\pi \nu \Delta_{FPI}\right)\right]}^{i}\right|}^{2}$.

Assume that the monochromatic parallel light beam converges at the image plane (x,y) after passing through the imaging system. For this image point, the incident light intensity is $B_{x,y}\left(\nu \right)$. Similar to the case without the FPI optical path, the OPD of the Sagnac interferometer is related to x, given by Δ=Dx/f. The OPD of the FPI is related to the field of view angle. For the image point at (x,y), the OPD of FPI is $\Delta_{FPI}=2nd\cos\theta_{x,y}$, where $\theta_{x,y}$ is the field of view angle at that point. FTIS usually has a small field of view, and $\theta_{x,y}$ of the entire field of view is close to 0. In $\Delta_{FPI,x,y}$, $\cos\theta_{x,y}$ is not sensitive to changes near 0. Therefore, $\Delta_{FPI,x,y}$ of the entire field of view can be approximately constant, meaning the FPI transmission spectrum in Equation (8) is $T_{FPI}\left(\nu \right)$. Therefore, the distribution of monochromatic light intensity at the image plane is as follows:

(8) $$I_{2}\left(x,y,\nu ,\Delta \right)=B_{x,y}\left(\nu \right)T_{FPI}\left(\nu \right)\left[1+\cos\left(2\pi \nu \Delta \right)\right],$$

This equation describes the interference light intensity distribution at the image plane of Detector 2 at a certain moment, corresponding to the single-frame data collected in Figure 3a–c. The process is the same as in Figure 3 based on the modulation scanning of TSMFTIS. The interferometric signals from the same target point are extracted and organized to obtain the interferogram of the target spectrum B(ν) after FPI modulation. At this point, the interferogram is only related to Δ, as follows:

(9) $$I_{2}\left(\Delta \right)=\int B\left(v\right)T_{FPI}\left(v\right)\cos\left(2\pi v\Delta \right)dv=FT\left[B\left(\nu \right)T_{FPI}\left(\nu \right)\right]=I_{0}\left(\Delta \right)*FT\left[T_{FPI}\left(\nu \right)\right].$$

where ∗ is the convolution operator. In Equation (9), $B\left(v\right)T_{FPI}\left(\nu \right)$ is regarded as a whole, where the role of the Sagnac interferometer is to perform an optical Fourier transform on them, and the role of the FPI is to modulate the incident spectrum B(ν) using $T_{FPI}$. Therefore, $I_{2}$ is the convolution of $I_{0}$ and $FT\left[T_{FPI}\left(\nu \right)\right]$.

### 2.3. Principle Analysis of Spectral Mixing in SMJI

**Figure 5:**
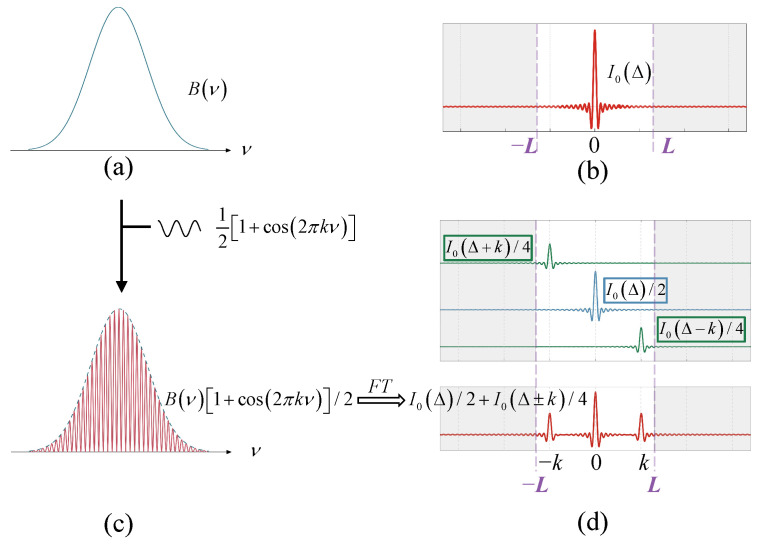
Schematic diagram of the spectrum mixing principle. (**a**) Object spectrum and (**b**) its interferogram; (**c**) cosine-modulated spectrum and (**d**) its interferogram.

Assume the target spectrum is B(ν) and its interferogram is $I_{0}\left(\Delta \right)$, as shown in Figure 5a,b. The two are in an FT relationship with each other. The interferometer has a maximum OPD of *L*, and the interference information of $I_{0}\left(\Delta \right)$ outside *L* will be lost. If a cosine spectrum with frequency *k* modulates B(ν), that is, $B\left(\nu \right)\left[1+\cos\left(2\pi k\nu \right)\right]/2$, as shown in Figure 5c,d, the interferogram of the modulated spectrum is as follows:

(10) $$I_{\cos}\left(\Delta \right)=I_{0}\left(\Delta \right)*FT\left\{\left[1+\cos\left(2\pi kv\right)\right]/2\right\}=\frac{1}{2}I_{0}\left(\Delta \right)+\frac{1}{4}I_{0}\left(\Delta -k\right)+\frac{1}{4}I_{0}\left(\Delta +k\right).$$

Therefore, the spectrum undergoes cosine modulation, and its effect on the interferogram is to generate two frequency shift components $I_{0}\left(\Delta \pm k\right)$, respectively, which are superimposed with $I_{0}\left(\Delta \right)$, as shown in Figure 5d. In this paper, we call this spectral mixing. In this process, the high-frequency interference information of $I_{0}\left(\Delta \pm k\right)$ is encoded into the low-frequency interference information of $I_{0}\left(\Delta \right)$.

From Equation (2), we can see that $T_{FPI}$ is composed of a fundamental wave and infinite high-order harmonics. Combining Equations (2) and (9), the $I_{2}\left(\Delta \right)$ obtained after B(ν) is modulated by $T_{FPI}$ and is expressed as follows:

(11) $$I_{2}\left(\Delta \right)=a_{0}I_{0}\left(\Delta \right)+\frac{1}{2}\sum_{m=1}^{\infty }a_{m}\left[I_{0}\left(\Delta -m\cdot \Delta_{FPI}\right)\right.\left.+I_{0}\left(\Delta +m\cdot \Delta_{FPI}\right)\right].$$

**Figure 6:**
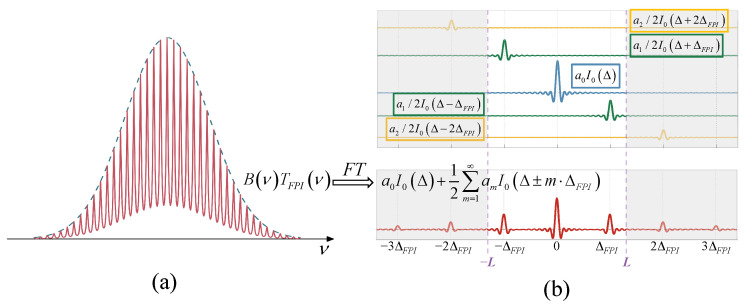
(**a**) Spectrum after FPI modulation; (**b**) schematic diagram of its interferogram.

Thus, the joint interference spectrum $B\left(\nu \right)T_{FPI}\left(\nu \right)$ and the interferogram $I_{2}\left(\Delta \right)$ can be obtained, as shown in Figure 6. In Figure 6b, $I_{2}\left(\Delta \right)$ can be considered as the superposition of the fundamental frequency component $a_{0}I_{0}\left(\Delta \right)$ and the frequency shift component $1/2a_{m}I_{0}\left(\Delta \pm m\cdot \Delta_{FPI}\right)$. Therefore, the complete interferogram $I_{2}\left(\Delta \right)$ will contain countless interference peaks gradually decaying at positions $\Delta =m\cdot \Delta_{FPI}$.

$I_{2}\left(\Delta \right)$ and $I_{1}\left(\Delta \right)$ share the same maximum OPD, *L*. The gray areas in the figure represent regions where the interference information exceeds *L*, and will thus be lost. The frequency shift component linearly combines $a_{0}I_{0}\left(\Delta \right)$Δ∈[−L,L] with the originally undetectable high-frequency information $a_{m}/2I_{0}\left(\Delta^{\prime }\right)$$\Delta^{\prime }\in \left[-L+m\Delta_{FPI},L+m\Delta_{FPI}\right]$. Therefore, the inversion algorithm can restore the high-frequency information to obtain a wider range interferogram and achieve spectral super-resolution.

## 3. Spectral Super-Resolution Inversion for SMJI-HI

### 3.1. Inversion Algorithm Calculation Principle

Based on Equation (11), to encode the high-frequency information of the m=1 level frequency shift component into the low-frequency information of the baseband component $I_{0}\left(\Delta \right)$, FPI needs to satisfy $\Delta_{FPI}=L$. Generally, the amplitude of the interferogram $I_{0}\left(\Delta \right)$ will decrease as Δ increases, and the coefficient am gradually decreases as m increases. Based on this, to simplify Equation (11), in the algorithm design, the energy of the components other than m=0,±1,2 within $\Delta \in \left[-L,L\right]$ can be ignored. $I_{2}\left(\Delta \right)$ can be expressed as follows:

(12) $$I_{2}\left(\Delta \right)=a_{0}I_{0}\left(\Delta \right)+\frac{a_{1}}{2}I_{0}\left(\Delta_{FPI}-\Delta \right)+\frac{a_{1}}{2}I_{0}\left(\Delta +\Delta_{FPI}\right)+\frac{a_{2}}{2}I_{0}\left(2\Delta_{FPI}-\Delta \right).$$

$I_{2}\left(\Delta \right)$ is a linear superposition of the fundamental frequency component $I_{0}\left(\Delta \right)$ and the frequency shift component. Therefore, it is necessary to establish a linear equation system of $I_{0}\left(\Delta \right)$ concerning $I_{1}\left(\Delta \right)$ and $I_{2}\left(\Delta \right)$. Based on Equation (12), a system of linear equations can be established as follows:

(13) $$\left[\begin{array}{c} I_{1}\\ I_{2} \end{array}\right]=\left[\begin{array}{cc} 1 & 0\\ A_{1} & A_{2} \end{array}\right]=AI_{0},$$

where $I_{1}$ and $I_{2}\in R^{N}$ are *N*-dimensional column vectors composed of $I_{1}\left(\Delta \right)$ and $I_{2}\left(\Delta \right)$ within Δ∈[0,L], respectively. $I_{0}\in R^{2N}$ is a 2N-dimensional column vector composed of $I_{0}\left(\Delta \right)$ within Δ∈[0,2L]. $A=\left[A_{1}\;A_{2}\right]\in R^{N\times 2N}$ is the coefficient matrix established based on Equation (12), and A is the overall coefficient matrix. Based on the calculated $I_{0}$, twofold spectral super-resolution can be achieved.

### 3.2. Interpolation of the Interferogram and Calculation of FPI’s OPD

In actual situations, *d* and cosθ of the FPI may experience errors due to environmental or alignment factors, causing $\Delta_{FPI}$ to deviate from the theoretical value. As analyzed in Section 2.3, $I_{2}\left(\Delta \right)$ exhibits interference peaks at $\Delta =m\cdot \Delta_{FPI}$. Based on this condition, $\Delta_{FPI}$ can be calculated from $I_{2}$. Therefore, it is necessary to calibrate the calculation of $\Delta_{FPI}$. During the FPI design phase, $\Delta =\Delta_{FPI}$ should be within the maximum OPD of the Sagnac interferometer. Based on Section 3.1, $\Delta_{FPI}$ should be slightly smaller than *L*, which can yield the interferogram of $a_{1}/2I_{0}\left(\Delta_{FPI}-\Delta \right)$ in the vicinity of $\Delta =\Delta_{FPI}$. Due to the uncertainty of $\Delta_{FPI}$, it cannot be precisely located at the sampling center of $I_{2}$. In Equation (13), it is necessary to establish a linear relationship between vectors $I_{2}$ and $I_{0}$. When $\Delta_{FPI}$ deviates from the sampling center, $I_{0}\left(\Delta_{FPI}-\Delta \right)$, $I_{0}\left(\Delta_{FPI}+\Delta \right)$, and $I_{0}\left(2\Delta_{FPI}-\Delta \right)$ in Equation (12) do not have corresponding points in the discrete vector $I_{0}$. Therefore, interpolation methods are needed to make the vectors $I_{1}$, $I_{2}$, and $I_{0}$ approximate continuous interferograms. As the sampling density increases, the effect of $\Delta_{FPI}$ deviating from the sampling center can also be neglected. Algorithms based on the interpolation between adjacent points will introduce computational errors. In this paper, $I_{1}$ and $I_{1}$ are first processed using an inverse Fourier transform (IFT) to obtain spectra $B_{1}$ and $B_{2}$; zeros are then padded to both ends of $B_{1}$ and $B_{2}$ before performing a Fourier transform (FT) to obtain the interpolated interferograms $I_{1}^{\prime }$ and $I_{2}^{\prime }$. The advantage of this interpolation method is that the interferometric intensity beyond the maximum wavenumber of the working spectral range is considered zero, and as a known condition for interpolation, this results in more minor interpolation errors.

**Figure 7:**
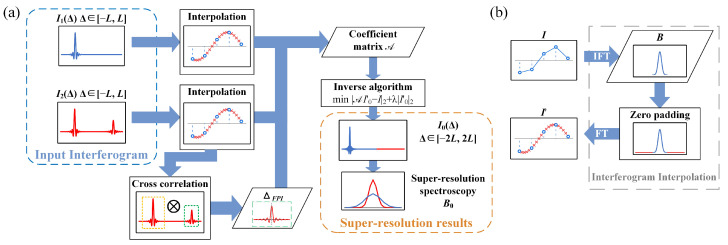
(**a**) Overall flowchart of the restoration algorithm; (**b**) flowchart of the interpolation algorithm.

According to Equation (11), $I_{2}\left(\Delta \right)$ can be regarded as composed of $a_{0}I_{0}\left(\Delta \right)$ and $a_{m}/2I_{0}\left(m\Delta_{FPI}\pm \Delta \right)$. The interferogram typically has higher amplitude at low frequencies, so $I_{0}\left(\Delta \right)$ generally has higher energy in the vicinity of U(0), where $U\left(\Delta \right)$ denotes the neighborhood of Δ. Therefore, in the neighborhoods U(0) and $U(\Delta_{FPI})$, $I_{2}^{\prime }\left[U\left(0\right)\right]\approx a_{0}I_{0}^{\prime }\left[U\left(0\right)\right]$ and $I_{2}^{\prime }\left[U\left(\Delta_{FPI}\right)\right]\approx a_{1}I_{0}^{\prime }\left[U\left(0\right)\right]$, as shown in Figure 6b. To calibrate $\Delta_{FPI}$, segments of the interferogram $I_{2}^{\prime }$ are extracted at $U\left(0\right)$ and $U\left(\Delta_{FPI}\right)$, obtaining $I_{2}^{\prime }\left[U\left(0\right)\right]$ and $I_{2}^{\prime }\left[U\left(\Delta_{FPI}\right)\right]$. By calculating the maximum value position of $I_{2}^{\prime }\left[U\left(0\right)\right]⊗I_{2}^{\prime }\left[U\left(\Delta_{FPI}\right)\right]$, the value of $\Delta_{FPI}$ can be determined, where ⊗ denotes the cross-correlation operator. Based on the determined $\Delta_{FPI}$ and Equation (13), a coefficient matrix A can be established for $I_{1}^{\prime }$ and $I_{2}^{\prime }$ concerning $I_{0}^{\prime }$. To reduce the ill-posedness of the problem, Equation (13) is converted into an optimization problem as follows:

(14) $$\min{\left|AI_{0}^{\prime }-I\right|}_{2}+\lambda {\left|I_{0}^{\prime }\right|}_{2},$$

where $I=\left[I_{1}^{\prime };I_{2}^{\prime }\right]$, ${\left|AI_{0}^{\prime }-I\right|}_{2}$ is the error term, $\lambda {\left|I_{0}^{\prime }\right|}_{2}$ is the regularization term to reduce ill-posedness and errors, and λ is the regularization coefficient used to balance the error term and the regularization term. The interferogram can be viewed as an oscillating curve around zero, so we chose the L2 norm of the entire interferogram as a regularization term to mitigate the ill-conditioned nature of the problem. The process of the spectral super-resolution algorithm is summarized in the Figure 7.

Since, in this paper, the light is split into only two paths after the Sagnac interferometer, and the FPI cannot change the interval *d* during the push-broom process, this enables a twofold spectral super-resolution. However, theoretically, more optical paths can be divided, each with different FPI spacings *d*. Using the same data processing method as for $I_{2}$ and establishing higher-dimensional linear equations can achieve triple or even higher spectral super-resolution. The challenge is that dividing into too many optical paths will lower the SNR and may introduce significant errors.

The above describes the process by which SMJI-HI enhances spectral resolution through spectral mixing via FPI, acquires interferograms using TSMFTIS, and recovers high-frequency information through inversion algorithms. In our previous research, we proposed MJI-HI as a super-resolution method specifically adapted for TMFTIS, discussed its key characteristics, and conducted comprehensive comparative analyses with other spectral super-resolution techniques. Existing methods achieving FTIS super-resolution through FPI (termed RS-HI in this paper) utilize tunable FPIs with adjustable cavity spacing to generate multiple narrow transmission peaks in the spectral dimension, thereby enabling finer resampling of FTIS sampling intervals. This approach achieves high super-resolution factors (7−17×). However, practical implementation challenges persist as follows: RS-HI requires high-precision FPIs with large cavity spacing (tuning step size 20–50 nm), stringent reflectivity requirements to maintain narrow transmission peaks, and exceptional mechanical stability during tuning. These constraints lead to excessive demands on FPI manufacturing precision and difficulties in achieving large apertures, contradicting TSMFTIS’s inherent advantages of high mechanical stability and large throughput. In contrast, SMJI-HI employs FPIs with fixed cavity spacing slightly below L/2 and lower reflectivity, significantly reducing manufacturing costs while enabling larger apertures and enhanced stability, thereby preserving TSMFTIS’s throughput and stability benefits.

Furthermore, since FPI cavity tuning modifies the periodic interval of transmission peaks rather than inducing global phase shifts, RS-HI requires interferogram acquisition quantities substantially exceeding the super-resolution factor to cover the full spectral range. Approximately 40–80 interferograms at different cavity spacings are needed per super-resolved spectrum, resulting in low data utilization efficiency and necessitating trade-offs between acquisition quantity and spectral coverage. For TMFTIS, such extensive acquisitions would prolong spectral measurement durations excessively. Implementation becomes fundamentally impractical for TSMFTIS, as traditional TSMFTIS employs push-broom scanning to sequentially sample target points across OPD dimensions. Incorporating RS-HI would require 40–80 signal acquisitions per pixel position with concurrent FPI tuning, followed by the extraction and integration of interferograms sharing identical FPI spacings—an unfeasible operational paradigm. Conversely, SMJI-HI enhances spectral resolution by indirectly extending maximum OPD, imposing no additional spectral range constraints beyond OPD sampling intervals. With higher data utilization efficiency, SMJI-HI achieves 2× super-resolution using two interferograms in this study, theoretically enabling *N*-fold resolution enhancement through *N* interferograms. This dramatic reduction in required interferograms facilitates implementation through optical path division rather than cavity-tuned FPIs. Consequently, SMJI-HI maintains compatibility with conventional TSMFTIS modulation mechanisms.

Based on this analysis, while RS-HI achieves high super-resolution factors, SMJI-HI demonstrates distinct advantages in FPI cost-effectiveness, data requirements, and control system complexity. It exhibits better compatibility with TSMFTIS regarding throughput, mechanical stability, and spectral coverage. Most critically, SMJI-HI is uniquely adapted to TSMFTIS’s specialized modulation mechanisms.

### 3.3. FPI Optical Defects and Algorithm Improvements

The above analysis is based on ideal FPI multi-beam interference. In reality, FPIs may have certain optical defects that could impact spectral super-resolution.

#### 3.3.1. OPD Offset

The frequency shift $\Delta_{FPI}$ primarily depends on the spacing *d* and the incident angle θ. Several factors can affect the spacing *d*, such as assembly tolerances, the thickness of the reflective layers, temperature variations, etc. Furthermore, the tilt of the FPI is a primary factor influencing θ. These defects and errors can lead to an offset in $\Delta_{FPI}$. Based on the interpolation and cross-correlation calculations described above, $\Delta_{FPI}$ calculation and calibration can be achieved.

#### 3.3.2. Frequency Shift Component Broadening

In practical machining and adjustment, the non-parallelism and non-flatness of the two reflecting surfaces of FPI introduce variations in optical path length at different transmission positions of FPI. The normalized cavity length errors G(Δd) and P(Δd) caused by non-parallelism and non-flatness are expressed as follows [36,37]:

(15) $$\begin{array}{cc} \; & G\left(\Delta d\right)=\frac{2}{\pi \alpha \rho }\sqrt{1-{\left(\Delta d/\alpha \rho \right)}^{2}},\\ & P\left(\Delta d\right)=\frac{1}{\sqrt{\pi }\Delta d_{D}}e^{-\frac{\Delta d^{2}}{\Delta d_{D}^{2}}}, \end{array}$$

where Δd represents the variation in FPI cavity length, α denotes misalignment, ρ is the radius of the circular aperture of the FPI, and $\Delta d_{D}$ represents the surface defect factor. It can be obtained that the full width at the half maximum (*FWHM*) of G(Δd) is $FWHM_{G}=1.5\alpha \rho$, and the *FWHM* of P(Δd) is $FWHM_{P}=2\sqrt{ln2}\Delta d_{D}$. The transmittance of the FPI spectrum considering non-parallelism and non-flatness is as follows:

(16) $$\begin{array}{cc} \; & T_{para}\left(\nu \right)=\int_{-\alpha \rho }^{+\alpha \rho }T_{FPI}\left(\nu ,d+\Delta d\right)G\left(\Delta d\right)d\left(\Delta d\right)/\int_{-\alpha \rho }^{+\alpha \rho }G\left(\Delta d\right)d\left(\Delta d\right),\\ \; & T_{flat}\left(\nu \right)=\int_{-\infty }^{\infty }T_{FPI}\left(\nu ,d+\Delta d\right)P\left(\Delta d\right)d\left(\Delta d\right)/\int_{-\infty }^{\infty }P\left(\Delta d\right)d\left(\Delta d\right). \end{array}$$

The spectrum and the interferogram are FT pairs. Substituting Equation (2) into Equation (16) and performing an FT yields the interferogram of the FPI transmission spectrum as follows:

(17) $$\begin{array}{cc} \; & {\tilde{T}}_{para}\left(\Delta \right)=a_{0}\delta \left(\Delta \right)+\sum_{m=1}^{\infty }\frac{a_{m}}{2\xi }\delta \left(\Delta \pm m\cdot \Delta_{FPI}\right)*G_{m}\left(\Delta \right),\\ \; & {\tilde{T}}_{flat}\left(\Delta \right)=a_{0}\delta \left(\Delta \right)+\sum_{m=1}^{\infty }\frac{a_{m}}{2\zeta }\delta \left(\Delta \pm m\cdot \Delta_{FPI}\right)*P_{m}\left(\Delta \right), \end{array}$$

where, ξ=∫G(Δd)d(Δd), ζ=∫P(Δd)d(Δd), $G_{m}\left(\Delta \right)=G\left(\Delta /2m\right)/2m$, $P_{m}\left(\Delta \right)=P\left(\Delta /2m\right)/2m$. The *FWHM* of $G_{m}\left(\Delta \right)$ and $P_{m}\left(\Delta \right)$ are 3mαρ and $4\sqrt{ln2}m\Delta d_{D}$, respectively. According to Equation (17), it can be seen that the frequency shift component of the FPI transmission spectrum’s interferogram changes from an δ function to a function broadened based on $G_{m}\left(\Delta \right)$ and $P_{m}\left(\Delta \right)$. The resulting interferogram is expressed as follows:

(18) $$\begin{array}{cc} \; & I_{FPI}\left(\Delta \right)=a_{0}I_{0}\left(\Delta \right)+\sum_{m=1}^{\infty }\frac{a_{m}}{2\xi }I_{0}\left(\Delta \pm m\cdot \Delta_{FPI}\right)*G_{m}\left(\Delta \right),\\ \; & I_{FPI}\left(\Delta \right)=a_{0}I_{0}\left(\Delta \right)+\sum_{m=1}^{\infty }\frac{a_{m}}{2\zeta }I_{0}\left(\Delta \pm m\cdot \Delta_{FPI}\right)*P_{m}\left(\Delta \right). \end{array}$$

Therefore, after passing through the FPI, the fundamental frequency component of the interferogram will not change due to this optical defect. In contrast, the frequency shift components of the interferogram will convolve with $G_{m}$ and $P_{m}$ and broaden. Its width increases *m*-fold with the order *m*, while its height decreases to 1/m with the order *m*.

#### 3.3.3. Algorithm Improvements

Based on the above analysis, the effects of non-parallelism and non-flatness of the FPI on the interferogram can be considered equivalent. By combining these two defects, the resulting interferogram is expressed as follows:

(19) $$I_{FPI}\left(\Delta \right)=a_{0}I_{0}\left(\Delta \right)+\sum_{m=1}^{\infty }\frac{a_{m}}{2\Omega }I_{0}\left(\Delta \pm m\cdot \Delta_{FPI}\right)*Defect_{m}\left(\Delta \right).$$

where $Defect_{m}\left(\Delta \right)=G_{m}\left(\Delta \right)*P_{m}\left(\Delta \right)$, $\Omega =\int G_{m}\left(\Delta \right)*P_{m}\left(\Delta \right)d\left(\Delta \right)$. If the width of the $Defect_{m}$ is relatively small, the effects of these two defects can be ignored; otherwise, the model needs to be improved to address these defects.

In the actual FPI manufacturing process, the non-parallelism and non-flatness of the fixed-interval FPI can usually be obtained through detection methods, so the theoretical value of $Defect_{m}\left(\Delta \right)$ can be considered known. $\left(a_{m}/2\Omega \right)*Defect_{m}\left(\Delta \right)$ can be written in vector form as $a_{m-Defect}\in R^{\varepsilon }$, where ε is the length of the $a_{m-Defect}$ vector, depending on the width of $Defect_{m}\left(\Delta \right)$. By replacing $a_{m}$ in the coefficient matrix A with the vector $a_{m-Defect}$, the broadening effect caused by $Defect_{m}$ can be simulated. Solving through Equation (13) can eliminate the broadening effect.

## 4. Experimental Simulation and Results Discussion

### 4.1. Simulation Conditions

In the simulation experiment, the spectral range is set to $1\times 10^{4}$∼$2\times 10^{4}$ ${\mathrm{cm}}^{-1}$; the maximum OPD of the Sagnac interferometer is L=57.6μm, resulting in a spectral resolution of Δν=1/(2L)=86.8 ${\mathrm{cm}}^{-1}$. Assume that a fixed air-spaced FPI is used, with a medium refractive index of n=1, a gap spacing of d=28.0μm, a FPI optical path difference of $\Delta_{FPI}=56.0\;\mu$m, a free spectral range (FSR) of 178.6μ${\mathrm{cm}}^{-1}$, and a reflectance of R=30%. The focal length of the converging system is f=85 mm, the detector has 512×512 pixels with a pixel size of 8.5μm, and the field of view is $2.93^{\circ }$ × $2.93^{\circ }$. The joint interference process is simulated by adding the parallel light complex amplitudes generated by double-beam interference and multi-beam interference with different optical paths. To simulate the effect of pixel size on the spatial modulation of TSMFTIS, each pixel is divided into 7×7 sub-pixels, sampled separately, and then, the weighted average of the samples is taken as the interferometric light intensity obtained by that pixel. The interferogram is generated by calculating and organizing the light intensity of all pixels in the X direction for the same input spectrum shown in Figure 3, simulating the temporal modulation of TSMFTIS.

**Figure 8:**
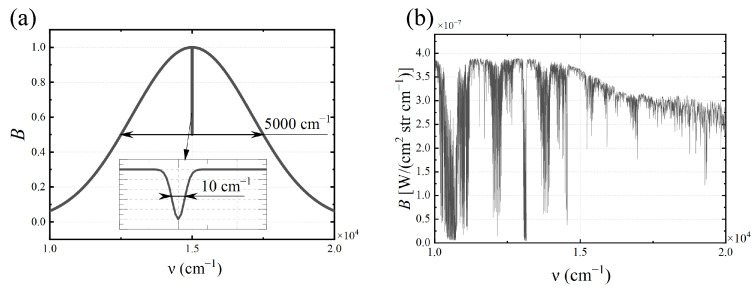
Input spectrum: (**a**) Gaussian function; (**b**) spectral data.

The simulation experiments use two different input spectra, as shown in Figure 8. These include the curve obtained by subtracting two Gaussian functions, respectively, with *FWHM* values of 5000 ${\mathrm{cm}}^{-1}$ and 10 ${\mathrm{cm}}^{-1}$, and spectral data calculated from atmospheric radiative transfer data.

In order to evaluate the simulation results more comprehensively and in greater detail, we quantitatively assess the interferograms and spectra using the following indicators:

Signal-to-Noise Ratio ($SNR_{I}$). We evaluate the inversion accuracy of the super-resolved interferograms using the $SNR_{I}$. The inversion algorithm of SMJI-HI mainly reconstructs the portion of the original interferogram $I_{0}\left(\Delta \right)$ in the range Δ∈[L,2L]. is the super-resolved inverted interferogram, and $I_{0}\left(\Delta \right)$ is the ideal interferogram obtained directly via the Fourier transform. The inversion algorithm of SMJI-HI mainly reconstructs the portion of the original interferogram $I_{0}\left(\Delta \right)$ in the range Δ∈[L,2L]. We calculate the $SNR_{I}$ based on the portion where Δ∈[L,2L] in both interferograms. Its expression is as follows:

(20) $$SNR_{I}\left(dB\right)=10\mathrm{lg}\left\{\frac{\sum {\left[I_{0}\left(\Delta \right)\right]}^{2}}{\sum {\left[I_{sup}\left(\Delta \right)-I_{0}\left(\Delta \right)\right]}^{2}}\right\}$$

Peak Signal-to-Noise Ratio (PSNR). The PSNR is used to evaluate the error between the spectrum B(ν) obtained by each imaging spectroscopic system in the simulation and the input spectrum $B_{0}\left(\nu \right)$. The higher the PSNR value, the closer it is to the input spectrum. Its expression is as follows:

(21) $$PSNR\left(dB\right)=10\mathrm{lg}\left\{\frac{MAX{\left[B_{0}\left(\nu \right)\right]}^{2}}{\sum_{i=1}^{N}{\left[B\left(\nu \right)-B_{0}\left(\nu \right)\right]}^{2}/N}\right\}$$

Structural Similarity Index (SSIM). The SSIM is commonly used to evaluate the structural similarity between signals, considering brightness, contrast, and structure. These factors collectively affect the perceived quality, and compared with the PSNR, the SSIM is more in line with the human visual perception of images or signals, thus generally providing an evaluation that better corresponds to actual visual quality. The SSIM is used to evaluate the structural similarity between B(ν) and $B_{0}\left(\nu \right)$. Let B(ν) be the signal *x* and $B_{0}\left(\nu \right)$ be the signal *y*; this is expressed as follows:

(22) $$SSIM=\frac{\left(2\mu_{x}\mu_{y}+c_{1}\right)\left(2\sigma_{xy}+c_{2}\right)}{\left(\mu_{x}^{2}+\mu_{y}^{2}+c_{1}\right)\left(\sigma_{x}^{2}+\sigma_{y}^{2}+c_{2}\right)}$$

Here, $\mu_{x}$ and $\mu_{y}$ are the means of signals *x* and *y*, $\sigma_{x}$ and $\sigma_{y}$ are the variances, $\sigma_{xy}$ is the covariance, and $c_{1}$ and $c_{2}$ are constants to avoid division by zero. The SSIM outputs a value between 0 and 1, with values closer to 1 indicating that the signals are more similar.

In addition, the number of iterations and the optimization time during the interferogram inversion process are used as indicators to evaluate the efficiency of the inversion algorithm. Based on experience with nonlinear solving, we employ the Infeasible path-following algorithm from the SDPT3 solver to optimize and solve the super-resolved interferogram $I_{sup}$.

### 4.2. Simulation Results

#### 4.2.1. Gaussian Input Spectrum

**Figure 9:**
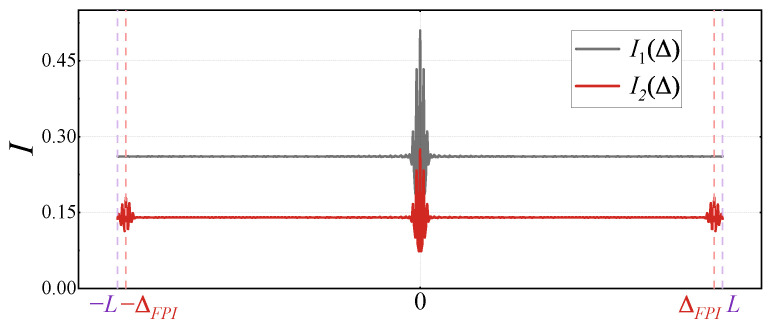
Interferograms $I_{1}$ and $I_{2}$.

Using Gaussian functions as input spectra, the interferograms $I_{1}\left(\Delta \right)$ and $I_{2}\left(\Delta \right)$ obtained by Detector 1 and 2 are shown in Figure 9, which corresponds to the non-shaded areas in Figure 6b. In $I_{1}\left(\Delta \right)$, interference peaks are only present at Δ=0. In $I_{2}\left(\Delta \right)$, interference peaks at $\Delta =\pm \Delta_{FPI}$ indicate the introduction of interference shift components due to the FPI. $I_{2}\left(\Delta \right)$ is the same as described in Figure 6, with interference peaks at $\Delta =\pm m\cdot \Delta_{FPI}$. Since $\Delta_{FPI}$ is slightly smaller than *L*, these interference peaks exceed the maximum OPD range and are lost. The high-frequency interference information of these frequency-shifted components is superimposed onto $I_{2}\left(\Delta \right)$ within the range of [−L,L]. Since this additional high-frequency information is ignored in Equation (12) when establishing the linear equations, it becomes one of the main sources of calculation errors.

**Figure 10:**
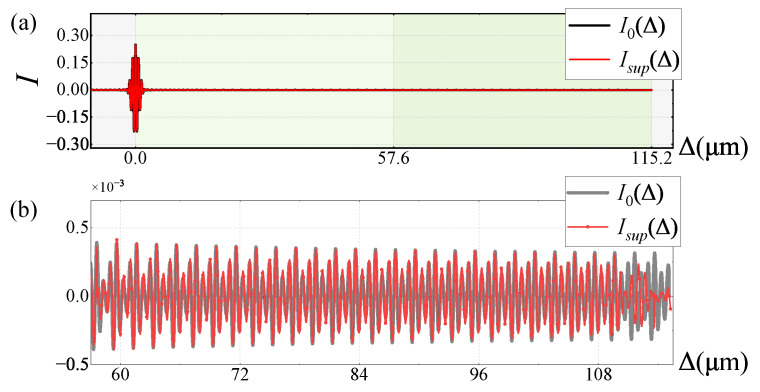
Interferogram of Gaussian input spectrum: (**a**) overall restoration result; (**b**) partial magnification of *L*∼2L.

Using the Gaussian function as the input spectra, the restored super-resolution interferogram $I_{sup}\left(\Delta \right)$ is compared with $I_{0}\left(\Delta \right)$ over the range $\Delta \in \left[-2L,2L\right]$, as shown in Figure 10. The results show that the high-frequency components of the interferogram are generally well restored. The main source of error at the $\Delta =2\Delta_{FPI}$ is the OPD positions near $\Delta =\Delta_{FPI}$ in $I_{2}\left(\Delta \right)$, where the computational error arises from separating the smaller amplitude $a_{1}/2I_{0}\left(\Delta_{FPI}+\Delta \right)$ from the larger amplitude $a_{1}/2I_{0}\left(\Delta_{FPI}-\Delta \right)$. It is necessary to add a regularization term to constrain the amplitude at this position. Consequently, the error at $\Delta =2\Delta_{FPI}$ is relatively smaller.

**Figure 11:**
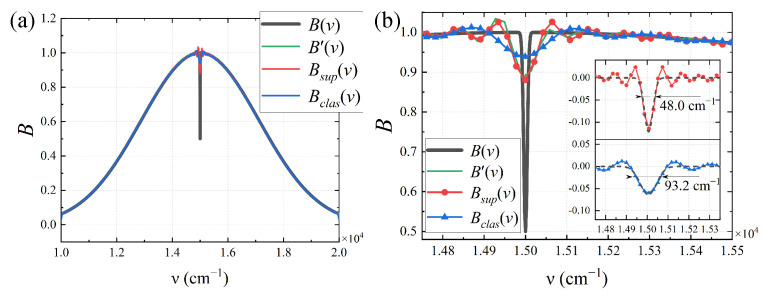
(**a**) Overall super-resolution spectral result of the Gaussian input spectrum; (**b**) partial magnification and the fitting and *FWHM* calculation of $B_{sup}$ and $B_{clas}$.

Using Gaussian functions with *FWHM* of 5000 ${\mathrm{cm}}^{-1}$ and 10 ${\mathrm{cm}}^{-1}$ to represent the low-frequency and high-frequency information of the spectrum, respectively, the super-resolution spectral results are shown in Figure 11. In the figure, B(ν) is the input spectrum; the red curve $B_{sup}\left(\nu \right)$ is the super-resolution spectrum obtained based on the super-resolution interferogram; the blue curve $B_{clas}\left(\nu \right)$ is the classical TSMFTIS spectrum obtained from $I_{0}\left(\Delta \right)$ with Δ∈[−L,L]; and the green curve $B^{\prime }\left(\nu \right)$ is the standard spectrum directly obtained from $I_{0}\left(\Delta \right)$ with Δ∈[−2L,2L]. According to the restoration results, $B_{sup}\left(\nu \right)$ matches the Gaussian function with a width of 7500 ${\mathrm{cm}}^{-1}$. For the high-frequency information represented by the Gaussian function with a width of 10 ${\mathrm{cm}}^{-1}$, it can be seen that $B_{sup}\left(\nu \right)$ has sharper peaks and is essentially consistent with $B^{\prime }\left(\nu \right)$. The *FWHM* results obtained from the Gaussian fitting of the spectra are shown in Figure 11b; the *FWHM* of $B_{clas}\left(\nu \right)$ is 93.2 ${\mathrm{cm}}^{-1}$, and the *FWHM* of $B_{sup}\left(\nu \right)$ is 48.0 ${\mathrm{cm}}^{-1}$. This demonstrates that the spectral resolution has been improved by nearly twofold.

#### 4.2.2. Spectral Data Input Spectrum

**Figure 12:**
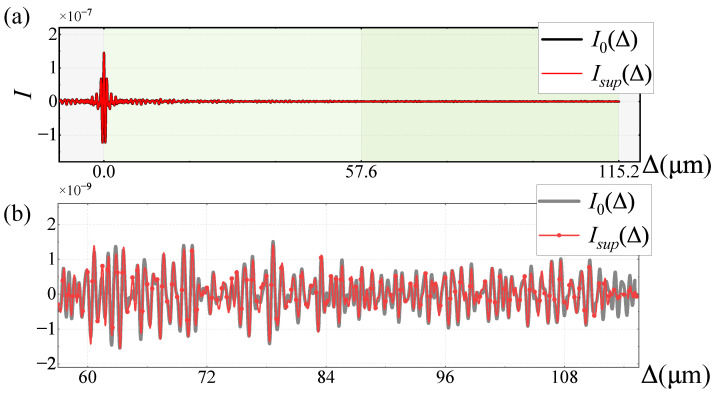
Interferogram of spectral data input spectrum: (**a**) overall restoration result; (**b**) partial magnification of *L*∼2L.

Using the spectral data as the input spectra, the restored super-resolution interferogram $I_{sup}\left(\Delta \right)$ is compared with $I_{0}\left(\Delta \right)$ over Δ∈[0,2L], as shown in Figure 12. The high-frequency interference information is overall well restored. Still, significant computational errors occur near $\Delta =2\Delta_{FPI}$, which need to be suppressed using regularization terms. The $SNR_{I}$ of $I_{sup}\left(\Delta \right)$ in the range of Δ∈[L,2L], as shown in Table 1, is 9.877 dB.

**Figure 13:**
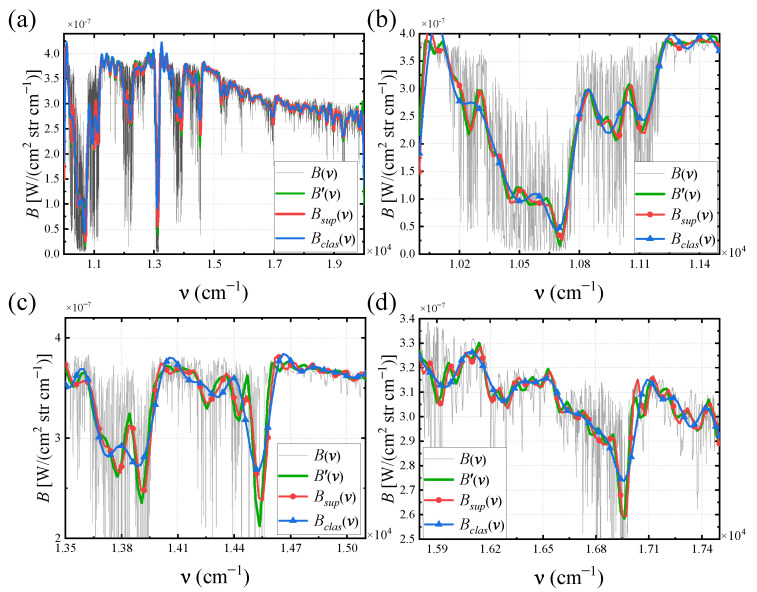
(**a**) Overall super-resolution spectral result of spectral data input spectrum; (**b**–**d**) partial magnifications.

The super-resolution spectrum $B_{sup}\left(\nu \right)$ is shown in Figure 13. Based on the numerical quantitative indicators proposed in Section 4.1, Table 1 shows the simulation results of SMJI-HI and TSMFTIS for the maximum OPD of *L* and 2L, respectively. From Figure 13 and the PSNR and SSIM in Table 1, it can be seen that $B_{sup}\left(\nu \right)$ is consistent with $B^{\prime }\left(\nu \right)$ and provides additional spectral details compared to $B_{clas}\left(\nu \right)$, indicating that the SMJI-HI is theoretically correct and feasible. In addition, the spectral data contains many absorption lines with widths much smaller than the sampling interval of $B_{sup}\left(\nu \right)$. This also indicates that when the interferogram contains a significant amount of high-frequency interference information beyond 2L, $B_{sup}\left(\nu \right)$ is less affected, demonstrating the high robustness of this method.

#### 4.2.3. Super-Resolution Results of the Entire Row Field

**Figure 14:**
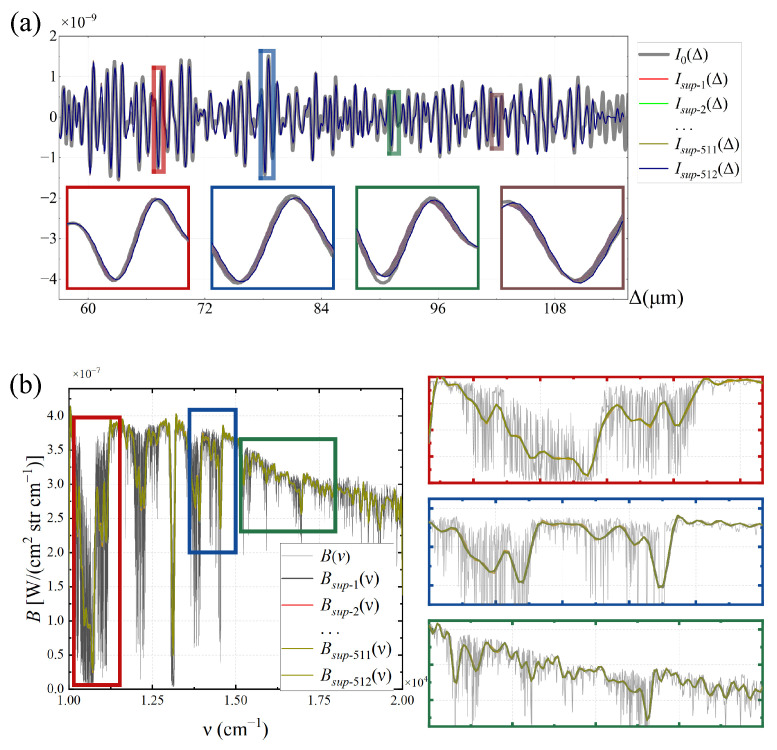
(**a**) Super-resolution interferogram and (**b**) super-resolution spectrum for each row of the field of view and its partial magnification.

TSMFTIS obtains the interferogram by traversing all pixels along the interference direction, which is the X direction in Figure 3. The above simulation analysis results are all from the center row of the field of view in the Y direction. To analyze the impact of different rows of the field of view, using spectral data as the input spectrum and keeping the same simulation conditions, the super-resolution interferogram obtained for each row of the field of view is shown in Figure 14.

Based on Figure 14a, it can be observed that the super-resolution interferograms obtained from each row of the field of view are largely overlapping overall, with minor differences visible upon close examination through partial magnification. The reason is that TSMFTIS has a small field of view angle, and $\Delta_{\mathrm{FPI}}$ is not sensitive to the field of view angle. This results in minor differences between the interferograms $I_{1}$ and $I_{2}$ of each row’s field of view, making the results quite similar. Performing FT on all super-resolution interferograms yields the super-resolution spectra, as shown in Figure 14b. Upon closer inspection through partial magnification, it can be seen that the super-resolution spectra from each field of view largely overlap with minor differences. As shown in Table 2, the $SNR_{I}$ of the interferogram $I_{sup}\left(\Delta \right)$ and the PSNR and SSIM of the super-resolved spectrum $B_{sup}\left(\nu \right)$ only undergo slight variations. Therefore, the super-resolution results obtained from different rows of the field of view with the same input spectrum are basically consistent, and the effect of the field of view angle can be neglected.

#### 4.2.4. Defect Simulation and Analysis

**Figure 15:**
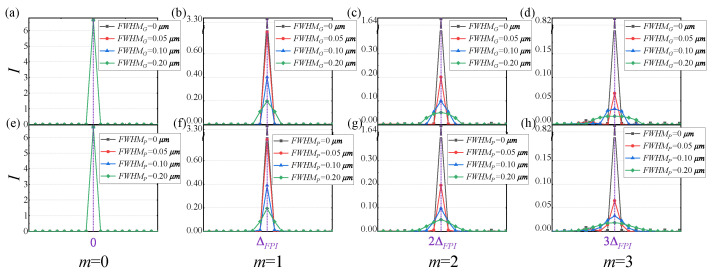
Frequency shift broadening caused by different degrees of non-parallelism for (**a**) m=0, (**b**) m=1, (**c**) m=2, (**d**) m=3; frequency shift broadening caused by different degrees of non-flatness for (**e**) m=0, (**f**) m=1, (**g**) m=2, (**h**) m=3.

Based on $T_{para}$ and $T_{flat}$, a simulation analysis of the FT of the FPI spectral transmittance under these two optical defect conditions is shown in Figure 15. The simulation analyzed the cases where the *FWH*$M_{G}$ and *FWH*$M_{P}$ are 0, 0.05, 0.10, and 0.20 μm, respectively. When the optical defects of the FPI are zero, its interferogram is consistent with Equation (11), with each frequency shift component being a δ function. When there is non-parallelism and non-flatness, the fundamental frequency component does not broaden, while frequency shift components convolve and broaden with $G_{m}$ and $P_{m}$. Moreover, as the order *m* increases, the broadening of the frequency shift components increases *m*-fold, and their height decreases to 1/m. As the optical defects increase, their broadening becomes wider. The simulation results are consistent with the analysis in Section 3.3.

**Figure 16:**
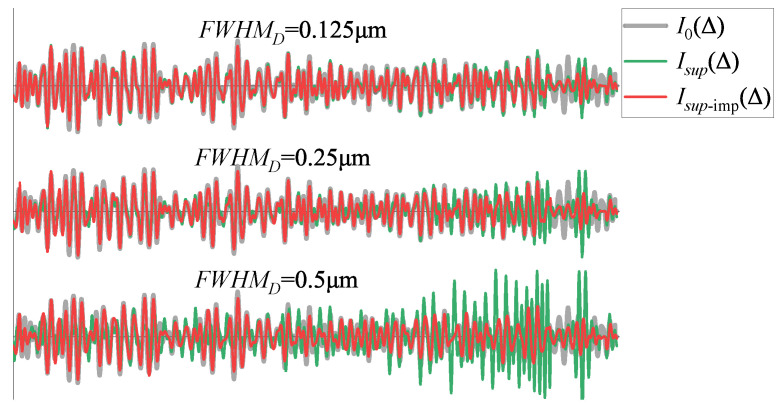
Interferogram restoration results for Defect1 with *FWH*$M_{D}$ values of 0.125 μm, 0.25 μm, and 0.5 μm.

Considering both types of optical defects, assuming the *FWHM* of $Defect_{1}$ in Equation (19) is 0.125, 0.25, and 0.5 μm, respectively, where the broadening due to non-parallelism and non-flatness each contributes 50%, the interferogram reconstruction is performed using both the improved and the unimproved algorithm from Section 3.3. The results shown as the red curve $I_{\sup-\mathrm{imp}}$ and the green curve $I_{\sup}$ in Figure 16. It can be seen that when the *FWH*$M_{D}$ is small, the results using the improved and unimproved algorithms are relatively close. As the *FWH*$M_{D}$ increases, the error in $I_{\sup}$ increases, while the result of $I_{\sup-\mathrm{imp}}$ changes less and remains more accurate. As shown in Table 3, under the unimproved inversion algorithm, both $SNR_{I}$ and PSNR decrease significantly as the $FWHM_{D}$ increases. It’s evident that the improved algorithm consistently delivers smaller errors. Therefore, the improved algorithm is feasible and effective. Moreover, the SSIM of the unimproved inversion algorithm increases as the error increases. This is because, as can be seen from Figure 13, $B_{0}$ exhibits a large number of absorption peaks; the increase in error causes the super-resolved spectrum $B_{\sup}$ produced by the unimproved inversion algorithm to oscillate, thereby making its structure more similar to that of $B_{0}$.

According to the Nyquist sampling theorem, the minimum sampling interval of the interferogram is $1/(2\nu_{max})$, which means the minimum sampling interval for this simulation is 0.25 μm. This can be used as a reference as follows: when the FWHM of the cavity length error function of the FPI’s optical defects is less than $1/(4\nu_{max})$, the broadening effect can be considered negligible. Currently, the manufacturing process of FPI is quite advanced. Non-parallelism usually meets the requirement of exceeding $1^{\prime \prime }$, and non-flatness is within tens of nanometers, typically better than this threshold. This issue can be addressed using the improved algorithm for cases with large broadening due to large clear apertures or other special circumstances.

## 5. Conclusions

This paper proposes a new FPI-based TSMFTIS spectral super-resolution technology, SMJI-HI. The spectral resolution of FTIS depends on the maximum OPD of the interferometer. TSMFTIS has a lower spectral resolution due to the limited size of the image plane. Due to its special modulation method, there is currently no method to significantly improve the spectral resolution. In information theory, TSMFTIS can be regarded as a spectral low-pass filter where interference information beyond the maximum OPD is lost. This paper introduces FPI into the system to surpass this low-pass system cutoff frequency, thereby enhancing spectral resolution. The role of FPI is that its transmitted spectrum can be seen as modulating waves to achieve spectral mixing, while TSMFTIS aims to obtain its interferogram. The interferogram after spectral mixing can be viewed as a linear combination of multiple displacement components of the original interferogram. This process shifts high-frequency interference information beyond the maximum OPD into the observable range. The inversion algorithm has been used to separate and reconstruct this high-frequency interference information, effectively extending the maximum OPD and achieving twofold spectral super-resolution. In theory, based on this method, it is possible to achieve threefold or even higher spectral super-resolution. However, this may lead to a reduction in energy during beam splitting.

This paper provides a detailed analysis of the joint interference intensity of the TSMFTIS and FPI based on interference theory and demonstrates the feasibility of the spectral super-resolution principle. Additionally, the algorithm was improved into an optimized mathematical model, with regularization terms set based on the characteristics of the interferogram to enhance its robustness. This paper conducts the simulation analysis and validation of this method using Gaussian spectra and spectral data as input spectra. The simulation results indicate that this method successfully achieves high-precision reconstruction of high-frequency interference information, resulting in significantly improved spectral resolution. Each row of the field of view can achieve spectral super-resolution without affecting the imaging. Furthermore, this paper discusses the effects of OPD offset and frequency shift component broadening introduced by optical defects in FPI. Based on these optical defects, adjustments and improvements to the algorithm are proposed. The FPI and TSMFTIS are both based on interference principles and exhibit the same characteristics in terms of field of view, spatial resolution, optical path length, and optical structure. This technology uses a fixed-spacing FPI, which offers advantages such as high throughput, compact structure, absence of mechanical moving parts, and low reflectance requirements. Additionally, it involves relatively straightforward spectral inversion calculations. While achieving improved spectral resolution at lower costs in weight, space, and price, it also ensures the advantages of high throughput, high stability, and the compact structure of TSMFTIS. Most importantly, there is no need for multiple FPI tuning, making it suitable for the interference principles and specialized modulation method of TSMFTIS. This technology achieves, for the first time, an enhancement in the spectral resolution of TSMFTIS, offering new approaches and methods for this research field.

## Figures and Tables

**Table 1:** SMJI-HI super-resolution interferogram and spectrum calculation results.

Hyperspectral Imaging System	${\mathrm{SNR}}_{I}$ (dB)	PSNR (dB)	SSIM	Iterations	Optimize Time (s)
FTIS (L)		18.107	0.5577		
FTIS (2L)		18.518	0.5660		
SMJI-HI	9.877	18.515	0.5635	36	14.8

**Table 2:** SMJI-HI super-resolution interferogram and spectrum calculation results of the entire row field.

Hyperspectral Imaging System	${\mathrm{SNR}}_{I}$ (dB)	PSNR (dB)	SSIM	Iterations	Optimize Time (s)
FTIS (L)		18.107	0.5577		
FTIS (2L)		18.518	0.5660		
SMJI-HI	8.263∼9.878	18.505∼18.515	0.5632∼0.5639	36∼37	12.1∼18.9

**Table 3:** SMJI-HI super-resolution interferogram and spectrum calculation results with and without the improved algorithm in the case of different degrees of broadening defects.

Hyperspectral Imaging System	${\mathrm{FWHM}}_{D}$	${\mathrm{SNR}}_{I}$ (dB)	PSNR (dB)	SSIM	Iterations	Optimize Time (s)
FTIS (L)			18.107	0.5577		
FTIS (2L)			18.518	0.5660		
SMJI-HI	0.125	9.102	18.502	0.5624	36	9.9
(improved	0.25	9.618	18.508	0.5619	36	12.2
algorithm)	0.5	8.256	18.493	0.5608	36	21.4
SMJI-HI	0.125	7.629	18.479	0.5652	37	16.6
(unimproved	0.25	3.187	18.312	0.5684	37	17.2
algorithm)	0.5	1.855	17.662	0.5761	37	16.5

## Data Availability

Data are contained within the article.

## Abbreviations

The following abbreviations are used in this manuscript:

FPI	Fabry–Pérot Interferometer
SMJI	Static Multi-Component Joint Interference Hyperspectral Imaging
FTIS	Fourier Transform Imaging Spectroscopy
TSMFTIS	Time–Space-Modulated FTIS
TMFTIS	Time-Modulated FTIS
SMFTIS	Space-Modulated FTIS
OPD	Optical Path Difference
